# A Narrative Review of Synaptic Transmission and Its Role in Neurological and Psychiatric Disorders: A Molecular Perspective

**DOI:** 10.7759/cureus.100649

**Published:** 2026-01-02

**Authors:** Nisar Ahmed, Haseeb Javaid Rather, Aditi Rana, Kaskar Alina Vasim, Komal Ravi Thaker, Dinesh Tripathi

**Affiliations:** 1 Department of Physiology, Shridevi Institute of Medical Sciences and Research Hospital, Tumkur, IND; 2 Department of Emergency Medicine, Pt. Madan Mohan Malaviya Hospital, New Delhi, IND; 3 Department of Obstetrics and Gynecology, Maharaja Tukojirao Holkar Women's Hospital, Mahatma Gandhi Memorial Medical College, Indore, IND; 4 Department of Medicine, Glocal University, Saharanpur, IND; 5 Department of Anesthesia, Dharmsinh Desai University, Dr. ND Desai Faculty of Medical Science and Research, Nadiad, IND; 6 Department of Biochemistry, Viraat Ramayan Institute of Medical Sciences, East Champaran, IND

**Keywords:** molecular neuroscience, neuropsychiatric disorders, precision medicine, synaptic transmission, synaptopathies

## Abstract

The ability of neurons to communicate via synapses is called synaptic transmission, and it is an essential process of brain functioning and plasticity. Its interference has been discovered as a common molecular trait in a broad range of neurological and psychiatric ailments. Nevertheless, in spite of increasing evidence within the disease context, the existing knowledge is still rather disunified, and the molecular processes are poorly incorporated into coherent, cross-disorder models. This narrative review addresses this gap by concisely synthesising recent advances in molecular genetics, synaptic proteomics, neuroimaging, and systems neuroscience to provide an integrated overview of synaptic dysfunction across neurological and psychiatric disorders. It reviews the role of the changes in vesicle trafficking, calcium dynamics, neurotransmitter receptor signalling, brain-derived neurotrophic factor (BDNF) action, and glia-mediated synaptic plasticity in the pathophysiology of conditions like schizophrenia, autism spectrum disorder (ASD), Alzheimer's disease (AD), epilepsy, major depressive disorder (MDD), and Parkinson's disease (PD). The emerging tools that have translational relevance, as pointed out by the review, include single-cell RNA sequencing, spatial proteomics, and synaptic positron emission tomography (PET) imaging, with the capabilities of providing disease-specific and patient-level insights into the pathology of synapses. This review establishes the convergence of the dysfunction, as well as therapeutic potential, through the presentation of a systems-level, cross-diagnostic framework at the level of the synapse. It ends with a prospective report of where precision medicine, development of new biomarkers, and lifespan research efforts are required to incorporate synaptic biology in translational neuroscience.

## Introduction and background

The basic mechanism by means of which neurons can exchange information is synaptic transmission, which occurs over specialised intercellular connections called synapses. This is a process of converting the electrical impulses into chemicals, which support the flow and alteration of information through neural circuits [1]. It forms an underlying basis to key brain processes, which include learning, memory, mood control, and motor coordination. This is choreographed by a set of rigidly controlled events comprising the docking of synaptic vesicles, neurotransmitter release, receptor activation, and termination of the signal, which are regulated by different sets of molecules [2]. The arsenal of scientific knowledge about the functioning of the synapses has increased to the degree that it is possible to speak about the use of electrophysiological, genetic, and imaging methods [3]. Studies of synaptic protein complexes have demonstrated that vesicle fusion at the presynaptic membrane is mediated by the soluble N-ethylmaleimide-sensitive factor attachment protein receptors (SNAREs), a group of proteins that drive membrane docking and fusion during neurotransmitter release [4]. N-ethylmaleimide-sensitive factor (NSF) facilitates this process by disassembling SNARE complexes after fusion, allowing vesicle recycling [5]. In addition, scaffolding proteins provide structural organisation by anchoring receptors and signalling molecules at synaptic sites, while neurotransmitter transporters regulate synaptic signalling by controlling the reuptake and clearance of neurotransmitters from the synaptic cleft [6]. These findings have helped to achieve a multi-level perspective of synaptic signalling with a range of subcellular nanostructures to systems-level neural plasticity.

Synaptic dysfunction has emerged as a very essential characteristic in the pathology of various neurological and psychiatric disorders [7]. Synaptopathies are disorders characterised by the dysfunction of synapses, wherein abnormalities in synaptic structure, signalling, or plasticity disrupt normal neuronal communication [8]. Imbalances in vesicle recycling, neurotransmitter release, receptor dynamics, or synaptic plasticity can destabilise or impair connections between neurons, and such alterations are collectively classified as synaptopathies [8]. A mutation in the synaptic adhesion molecule encoding genes has been reported in the autism spectrum disorder (ASD) to be associated with abnormal synapse formation and connection [6]. Schizophrenia is linked with hypofunction of N-methyl-D-aspartate (NMDA)-type glutamate receptors and changes in the postsynaptic density proteins, leading to the disorganisation of the cortex and the loss of cognitive functions [7]. Loss and dysfunction in the synapses are also early and predictive of neurodegenerative diseases. Synaptic loss in Alzheimer's disease (AD) is frequently preceded by the covert death of neurons and is more directly linked to cognitive losses than is proteinaceous aggregate deposition [8]. In Parkinson's, destruction of dopaminergic synapses of the motor control pathways disrupts movement and may cause non-motor symptoms, including cognitive impairment and mood disorders [9]. In epilepsy, an imbalance in the excitatory-inhibitory transmission balance, commonly reflecting abnormalities of GABAergic signalling or synaptic vesicle cycling, causes hyperexcitation and inappropriate neural synchronisation [10,11].

Beyond structural degeneration, alterations in synaptic plasticity are increasingly implicated in affective disorders, particularly major depressive disorder (MDD) [12,13]. In MDD, loss of synaptic connections and reduced synaptic plasticity have been consistently observed in key limbic and cortical regions, including the hippocampus, prefrontal cortex, anterior cingulate cortex, and amygdala, which are critically involved in mood regulation, emotional processing, and cognitive control [14]. The rapid-acting antidepressants have been found to increase synaptic strength, α-amino-3-hydroxy-5-methyl-4-isoxazolepropionic acid (AMPA) receptor-mediated signalling, and activity-dependent remodelling of dendritic spines, which was found to be clinically effective [4]. This suggests that dysfunction in the area of synapses may underlie both cognitive and emotional symptoms across the disease spectrum. Genomic and proteomic studies of large-scale populations have also highlighted the fact that the synaptic components are at the centre stage when it comes to disease susceptibility [5]. Several proteins encoded by risk loci identified in genome-wide association studies are integral components of synaptic vesicle cycling, a process that governs vesicle docking, fusion, endocytosis, and recycling; receptor trafficking, which regulates the insertion, removal, and lateral movement of neurotransmitter receptors at synaptic membranes; and intracellular signalling cascades that transmit synaptic activity to downstream molecular pathways influencing plasticity and gene expression [2,8]. In line with that, transcriptomic studies have also found the systematic downregulation of synapse-related gene networks in the brain areas affected in several disorders [6]. These lines of evidence converge on growing interest in synapse-directed therapeutic strategies, including receptor modulators, stabilisation of synaptic proteins, and neuromodulatory approaches targeting specific neural circuits [2,14].

**Figure 1:**
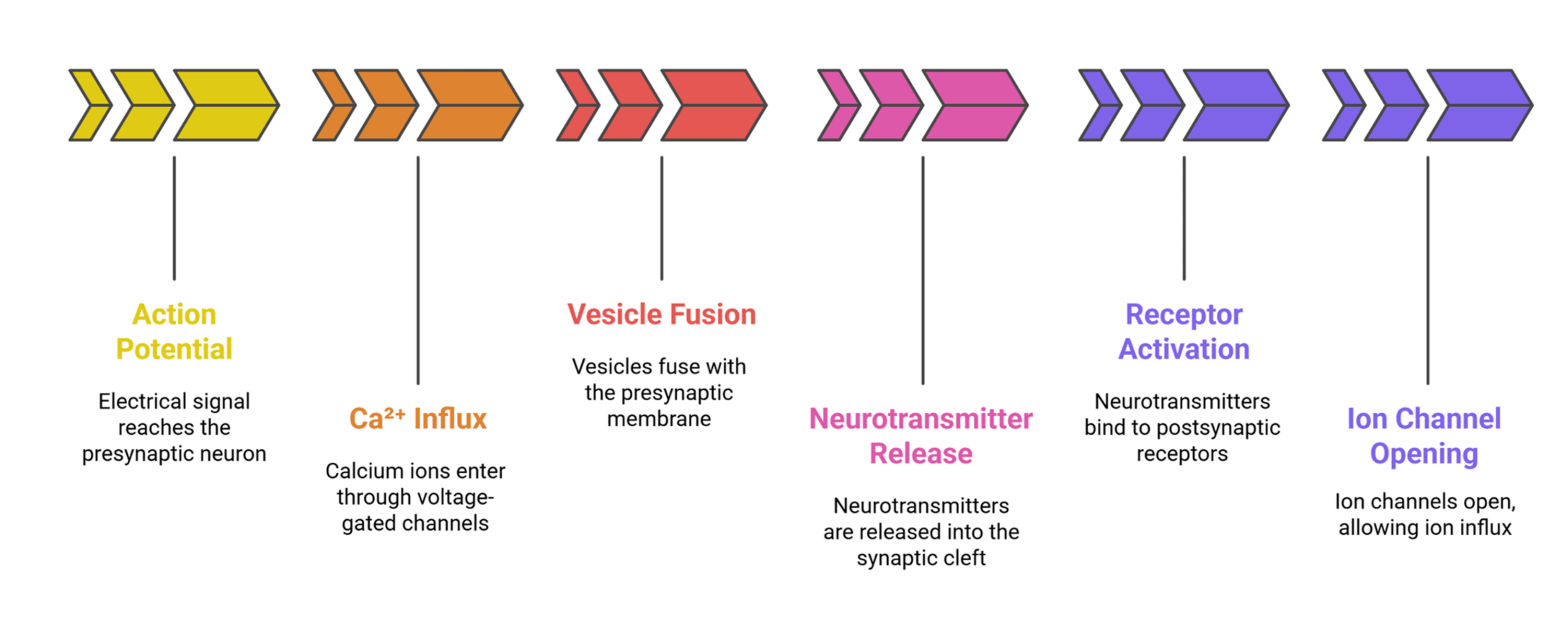
Sequential steps in synaptic transmission. The figure was created by the authors.

Figure 1 depicts the six key stages of synaptic transmission: action potential arrival, Ca²⁺ influx, vesicle fusion, neurotransmitter release, receptor activation, and ion channel opening.

This narrative review provides an in-depth analysis of the molecular basis of synaptic transmission and its contribution to the pathophysiology of major neurological and psychiatric disorders. It focuses on disease-associated alterations in key synaptic processes, including neurotransmitter release, vesicle fusion, receptor modulation, and synaptic plasticity. The review discusses disorder-specific synaptic mechanisms in ASD, Parkinson’s disease (PD), epilepsy, MDD, schizophrenia, and AD, and highlights recent advances in molecular profiling, neuroimaging techniques, and approaches for synaptic manipulation. By integrating mechanistic insights with clinical implications, this narrative review aims to elucidate how synaptic dysfunction underlies brain disorders and to emphasise synaptic pathways as promising therapeutic targets.

## Review

Methodology

This narrative review was conducted through a broad and flexible survey of peer-reviewed literature addressing synaptic transmission and synaptic dysfunction in neurological and psychiatric disorders. Relevant publications were identified using major scientific databases, including PubMed, Scopus, Web of Science, and Google Scholar. Search terms included combinations of keywords such as synaptic transmission, synaptic plasticity, synaptopathies, neurological disorders, psychiatric disorders, vesicle cycling, receptor signalling, and neuroinflammation. Literature published mainly over the past 10 years was emphasised to capture contemporary advances, while selected earlier studies were included to provide essential conceptual background.

Studies were included if they contributed to the understanding of synaptic mechanisms at the molecular, cellular, or systems level in the context of neurological or psychiatric disorders. Articles were excluded if they lacked relevance to synaptic function or dysfunction, focused solely on non-neuronal or non-synaptic processes, or did not provide mechanistic or translational insight. Consistent with the narrative nature of this review, no formal systematic selection process or quantitative meta-analysis was applied.

Molecular architecture of synaptic transmission

The synaptic transmission starts with the presynaptic terminal, in which the neurotransmitters are packed into the synaptic vesicles and placed at the active zones, ready to be released. The active zone has well-organised protein complexes that coordinate docking, priming, and fusion of vesicles in response to an arriving action potential [11]. When depolarisation occurs, voltage-gated calcium channels open, which are concentrated in the active zone, and hence calcium ions intrude into the cytosol of the presynaptic cell [12]. This calcium elevation is necessary in the activation of exocytosis, which is through binding with calcium sensors located on the membrane of the vesicle [13]. Vesicle fusion is orchestrated by the SNARE complex, a conserved protein assembly that drives membrane docking and fusion during neurotransmitter release. The SNARE complex consists of the vesicle-associated protein synaptobrevin (v-SNARE) and the plasma membrane proteins syntaxin-1 and synaptosomal-associated protein 25 (SNAP-25; t-SNAREs), which assemble into a stable helical bundle that pulls the synaptic vesicle into close apposition with the presynaptic membrane, thereby enabling fusion [14]. Synaptotagmin is the major calcium sensor that, upon binding calcium, triggers fusion at this critical level [15]. Following vesicle fusion, neurotransmitters are released into the synaptic cleft, where they rapidly diffuse and bind to postsynaptic receptors, initiating signal transmission [11].

A synaptic cleft is more than a gap. It has extracellular matrix proteins, adhesion molecules, and enzymes that regulate the dispersion and elimination of neurotransmitters [16]. The postsynaptic membrane, especially at excitatory synapses, has a thick cluster of proteins called the postsynaptic density (PSD). The area has both ionotropic and metabotropic receptors, plus the scaffolding proteins, such as postsynaptic density protein 95 (PSD-95), which fix the receptors at specific locations [17]. Following neurotransmitter release, synaptic vesicle membranes are retrieved through endocytosis, a cellular process by which portions of the plasma membrane are internalised to reform vesicles for reuse. The predominant mechanism is clathrin-mediated endocytosis, in which the protein clathrin assembles into a coated pit that invaginates and pinches off from the presynaptic membrane, enabling efficient vesicle recovery and recycling. In addition to this canonical pathway, ultrafast and clathrin-independent endocytic mechanisms also operate, particularly during periods of high-frequency synaptic activity, to maintain synaptic transmission [18]. The retrieved vesicles are refilled with neurotransmitters and incorporated into distinct synaptic vesicle pools, which represent functionally defined groups of vesicles available for future release. These pools include the readily releasable pool, which is immediately available for exocytosis; the recycling pool, which sustains neurotransmission during moderate activity; and the reserve pool, which is mobilised during prolonged or intense synaptic stimulation. Such pools are divided into readily releasable, recycling, and reserve ones, each of which is controlled with the help of specific molecular mechanisms [19].

The release of vesicles is time and probability-dependent, according to their interaction with the calcium channel position, availability of the SNARE complex, and state of vesicle priming. Alteration of any of these elements can result in synaptic delay, reduced reliability of transmission, or neurotransmission failure altogether [20]. In this way, the molecular architecture of the synapse is exquisitely organised to ensure rapid, efficient, and adaptable communication between neurons [11].

Neurotransmitter systems and synaptic plasticity

The neurotransmitters used in the synaptic transmission are classified as excitatory, inhibitory, or modulatory broadly. Glutamate, the primary excitatory neurotransmitter in the central nervous system, acts through NMDA receptors and AMPA [21]. The gamma-aminobutyric acid (GABA) is the key inhibitory neurotransmitter that has an effect on GABAA and GABAB receptors, stabilising membrane potentials and decreasing neuronal firing [22]. Acetylcholine plays a critical role in attention and learning through the activation of nicotinic and muscarinic receptors [11]. Dopamine plays an important role in motor control and reward signalling and works via several receptor subtypes, which activate cyclic AMP pathways [23]. Other neuromodulators include serotonin and norepinephrine, which influence mood, arousal, and sensory processing [11,20].

Synaptic strength is dynamic and varies with neuronal activity. Long-term potentiation (LTP) is a sustained increase in synaptic strength, reflecting an activity-dependent enhancement in the efficiency of synaptic transmission, and is typically induced by high-frequency stimulation. In contrast, long-term depression (LTD) is a persistent reduction in synaptic strength that occurs following specific patterns of low-frequency stimulation or reduced calcium signalling, serving as a complementary mechanism for weakening synaptic connections. By activating NMDA receptors, triggering intracellular kinases, and increasing AMPA receptor insertion into the postsynaptic membrane, glutamatergic synapses create LTP [24]. Conversely, LTD decreases strength at the synapses and is usually caused by reduced calcium influx. LTD could be through internalisation of receptors, changes in receptor phosphorylation, or through the induction of phosphatases that counteract the effects of LTP [13]. These opposing mechanisms allow synapses to bidirectionally modify their strength, a process that is essential for memory encoding, refinement of sensory maps, and maintenance of synaptic homeostasis [19].

Synaptic plasticity refers to the ability of synapses to modify their strength or efficacy in response to patterns of neuronal activity and is a fundamental mechanism underlying learning, memory, and adaptive behaviour. Plasticity can be broadly categorised into long-term and short-term forms based on the duration of synaptic change. Short-term synaptic plasticity is a transient, activity-dependent modification of synaptic strength that occurs over milliseconds to seconds and is primarily mediated by presynaptic mechanisms. It includes paired-pulse facilitation, defined as a temporary increase in synaptic response caused by residual presynaptic calcium following closely spaced stimuli, and paired-pulse depression, characterised by a reduced synaptic response due to depletion of readily releasable synaptic vesicles. These forms of short-term plasticity regulate information transfer and filtering at synapses and depend on residual calcium levels and vesicle availability [14]. Further, inhibitory synapses also display their type of plasticity, i.e., inhibitory LTP and LTD, contributing towards control of the excitatory-inhibitory balance and synchronisation of the network activity [17]. Neuromodulators also influence the plasticity of the synapses through modulation of the induction threshold of either LTP or LTD. As an example, LTP can be facilitated in some cortical regions by the release of dopamine [22], and acetylcholine plastically regulates the hippocampus [22]. These influences show that synaptic modification is influenced not only by the local spike timing but also by its contingent nature. Metaplasticity refers to the regulation of synaptic plasticity itself, whereby prior synaptic activity modifies the threshold and direction of subsequent plastic changes, thereby preventing the saturation of LTP or LTD and maintaining synaptic adaptability. It guarantees that plasticity would be dynamic and adjustable and not stationary or unidirectional [19]. This level of control is especially important in learning contexts, where continual reassessment and reorganisation of synaptic priorities are required to support adaptive information processing [19]. Table 1 summarises key neurotransmitters, their types, receptors, and associated plasticity mechanisms, highlighting their effects on synaptic strength and function.

**Table 1:** Neurotransmitters and their roles in synaptic plasticity. AMPA: α-amino-3-hydroxy-5-methyl-4-isoxazolepropionic acid; NMDA: N-methyl-D-aspartate; LTP: long-term potentiation; LTD: long-term depression; GABAA: gamma-aminobutyric acid type A receptor; GABAB: gamma-aminobutyric acid type B receptor; 5-HT: 5-hydroxytryptamine (serotonin); GPCR: G protein–coupled receptor; D1–D5: dopamine receptor subtypes 1 to 5.

Neurotransmitter	Type	Receptors	Associated plasticity	Mechanism/effect
Glutamate	Excitatory	AMPA, NMDA	LTP, LTD	LTP: Ca²⁺ influx via NMDA → AMPA insertion. LTD: low Ca²⁺ → AMPA internalisation
GABA	Inhibitory	GABAA, GABAB	Inhibitory LTP/LTD	Regulates excitatory-inhibitory balance; stabilises membrane potential
Acetylcholine	Modulatory	Nicotinic, Muscarinic	Metaplasticity	Enhances cortical plasticity; modulates hippocampal learning circuits
Dopamine	Modulatory	D1–D5 (GPCRs)	Facilitated LTP	Increases LTP probability; modulates reward-linked synaptic changes
Serotonin	Modulatory	5-HT receptors (multiple subtypes)	Neuromodulatory plasticity	Alters plasticity thresholds; affects mood and emotional learning
Norepinephrine	Modulatory	α, β adrenergic receptors	Neuromodulatory plasticity	Modifies synaptic responsiveness to stimuli; enhances arousal-based learning

Synaptic dysfunction in neurodevelopmental disorders

Neurodevelopmental disorders are usually caused by the interference with synaptic transmission at critical stages of brain development. Such dysfunctions may entail structural, functional, or molecular defects of synaptic proteins that govern transmission, plasticity, and connection [20]. ASD is prominently associated with abnormalities in synaptic adhesion molecules, particularly neurexins and neuroligins, which are transsynaptic proteins that span the presynaptic and postsynaptic membranes to align neurotransmitter release sites with postsynaptic receptors. Neurexins are primarily presynaptic organisers that regulate neurotransmitter release probability, whereas neuroligins are postsynaptic partners that influence synapse specification and maturation. Defects in these molecules disrupt the balanced development of excitatory and inhibitory synapses, leading to an altered excitation-inhibition (E/I) ratio that impairs circuit stability and information processing in ASD [11]. Such adhesion proteins span the presynaptic and postsynaptic membranes and align neurotransmitter release sites with postsynaptic receptors. Mutations in neuroligins can increase inhibitory synapse formation, thereby altering the E/I ratio, the balance between excitatory and inhibitory synaptic inputs and disrupting local circuit dynamics [12]. Moreover, deletions in neurexin genes lower the probability of the presynaptic release and disrupt the development of the synaptic contacts [13].

The most common inherited factor, fragile X syndrome, causes the loss of fragile X mental retardation protein (FMRP). FMRP controls synaptic protein translation in dendrites, and its loss results in an unregulated protein synthesis [14]. This encompasses overstimulated signalling using group I metabotropic glutamate receptors (mGluR5) that enhances the overproduction of LTD and impairs the synaptic connection [15]. The neurodevelopmental disorders also demonstrate a deficiency of activity-dependent plasticity. Reduced LTP, increased LTD, and dendritic spine morphology, including immature or elongated spines, are common in animal models [16]. Such phenotypes imply that the synaptic circuits are not sufficiently developed by experience and can have a juvenile structure during adulthood.

Imbalance of E/I is common in most neurodevelopmental disorders. Hyperexcitable cortical circuits have been reported in human electroencephalography (EEG) recordings and in mouse ASD models [17]. The alterations can be the result of the decrease in GABA-mediated (GABAergic) inhibition, the change in glutamate release, or the inability to regulate chloride in developing neurons [18]. In addition to the neurons, the glial cells are essential in the formation, maintenance, and destruction of synapses. The microglia proactively remove the surplus synapses in the course of development, and the guidance of the immune-related signals. The imbalance of these pathways may result in an overgrowth of synapses or retention of weak synapses, thus causing circuit noise and poor information processing [19].

**Figure 2:**
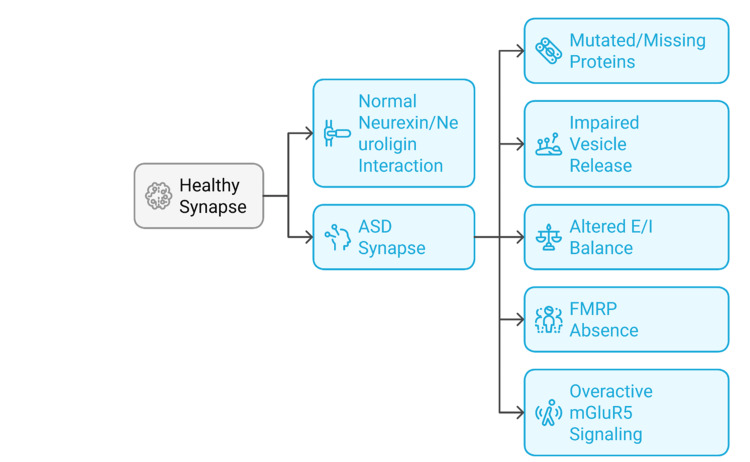
Synaptic alterations in ASD. ASD: autism spectrum disorder; E/I: excitation–inhibition; FMRP: fragile X mental retardation protein; mGluR5: metabotropic glutamate receptor 5. The figure was created by the authors.

Genetic predisposition and genetic vulnerabilities can be combined with environmental risk factors, including maternal immune activation, prenatal exposure to toxins, or stress in early life, and promote synaptic dysfunction [20]. Such factors can disrupt synaptogenesis, astrocyte signalling, and neuromodulatory system development, thereby contributing to long-term neurodevelopmental deficits [17,19,20]. Collectively, these findings support the view that synaptic dysfunction represents a key and unifying pathological process underlying neurodevelopmental disorders [8,16]. Accordingly, therapeutic strategies aimed at restoring synaptic protein expression, normalising receptor activity, and re-establishing E/I balance have been proposed and explored as potential treatment approaches [2,14]. Figure 2 illustrates synaptic abnormalities observed in individuals with ASD compared with healthy synapses, including mutated or deficient synaptic proteins and disrupted neurexin-neuroligin interactions that impair synapse formation [11,25], reduced presynaptic vesicle release probability [13], altered E/I balance due to disproportionate inhibitory or excitatory synapse development [12,17], absence or dysfunction of FMRP leading to dysregulated synaptic protein synthesis [14], and overactivation of group I metabotropic glutamate receptor 5 (mGluR5) signalling, which contributes to excessive LTD and synaptic instability [15].

Synaptic abnormalities in schizophrenia

Schizophrenia is a neuropsychiatric illness that entails cognitive, perceptual, and affective disorders. There is accumulating evidence that its underlying pathophysiology plays a central role in disturbances in synapses [25]. Hypofunction of NMDA receptors is one of the most repeatable findings, and these are glutamate-gated ion channels that play a central role in synaptic plasticity and neural circuit modulation [26]. Antagonists of NMDA receptors, such as ketamine and phencyclidine, have been shown to induce schizophrenia-like features in both animal models and humans [27]. The decreased activity of NMDA receptors will have downstream effects on calcium signalling, which damages the LTP and decreases the stability of dendritic spines in cortical and hippocampal neurons [28]. According to postmortem research among schizophrenic patients, the NMDA receptor subunits show a reduction in their expression and localisation at the synaptic site [29]. These impairments disrupt neural synchrony and working memory, cognitive domains that are prominently affected in schizophrenia [28].

In addition to disruptions in glutamatergic signalling, abnormalities in the disrupted-in-schizophrenia-1 (DISC1) gene, a schizophrenia susceptibility gene involved in synaptic development, neuronal migration, and intracellular signalling, contribute to impaired synaptic maturation and function. Alterations in DISC1 expression or function have been associated with disrupted cortical circuitry and cognitive deficits characteristic of schizophrenia [30]. DISC1 modulates various intracellular signalling pathways that deal with cytoskeletal assembly, vesicle transport, and postsynaptic signalling [31]. Interference of these interactions may result in shifted dendritic arborization and diminished synaptic relations. The other pathway that has also been found to contribute to schizophrenia is the neuregulin-1 (NRG1)/Erb-B2 receptor tyrosine kinase 4 (ErbB4) signalling pathway. NRG1 binds to the ErbB4 receptor of GABAergic interneurons, which affects their maturation and operation [32]. Alterations in the levels of NRG1 or ErbB4 are linked to any impairment in inhibition control and disrupted cortical oscillations that form the foundation of sensory gating and attention control [33].

The other important point is the PSD protein that fixes receptors and assembles signalling molecules of the synapse. In the patient with schizophrenia, the reductions in major scaffolding protein PSD-95 have been observed, especially in the dorsolateral prefrontal cortex [34]. These modifications destabilise the receptor and interfere with signal transfer in the excitatory synapses. In general, convergence of molecular, genetic, and physiological findings underlies a model where a role in both symptomatology and the development of schizophrenia plays synaptic dysfunction, which is both excitatory and inhibitory [35]. NMDA receptor-enhancing interventions, restoration of synaptic scaffolding protein networks, and modulation of ErbB4 signalling are currently being investigated as potential therapeutic approaches for schizophrenia [2,26].

Synaptic degeneration in Alzheimer’s disease

Amnesia and mental decline are hallmarks of AD, a degenerative neurological illness. Although the death of neurons is characteristic of late-stage AD, the degeneration of synapses is much more proximal, and it precedes cognitive dysfunction by far [25]. At the initial phases of the illness, synapses are lost in large quantities in the hippocampus and neocortex, affecting the network communication and functional connectivity [26]. Synaptic toxicity is mediated by amyloid-beta (Aβ) peptides, which are generated through the proteolytic cleavage of amyloid precursor protein and contribute to early synaptic dysfunction in AD [27]. Synaptic signalling is disrupted by soluble A oligomers by disrupting receptor trafficking, enhancing calcium dysregulation, and decreasing dendritic spine density [27]. The oligomers may inhibit the NMDA and AMPA receptors and cause a lack of excitatory drive and changes in plasticity [28].

Tau is a microtubule-associated protein that stabilises neuronal cytoskeletal structure and supports axonal transport. When tau becomes hyperphosphorylated, it dissociates from microtubules, mislocalises to dendrites, and disrupts synaptic signalling, thereby contributing to synaptic deterioration. In parallel, pro-inflammatory cytokines (small immune signalling molecules) and reactive oxygen species (chemically reactive oxygen-containing molecules) released during neuroinflammation further exacerbate synaptic damage by promoting oxidative stress and impairing synaptic function. Tau in its pathological state dissociates with microtubules and becomes accumulated in dendrites, disrupting postsynaptic receptors' localisation and mitochondrial transport [29]. This mislocalisation inhibits synapses' energy balance and makes them susceptible to oxidative stress [30]. Neuroinflammation plays a compounding role in synaptic degeneration. When activated, microglia, the brain’s resident immune cells, release cytokines, which are small inflammatory signalling proteins, and reactive oxygen species, which are highly reactive oxygen-derived molecules that induce oxidative stress and damage cellular components, including synapses. Excessive microglial activation can also lead to abnormal synaptic pruning through complement-mediated pathways, resulting in the removal of functional synapses beyond physiological levels and thereby contributing to synaptic loss and cognitive impairment [31]. There is also a loss of astrocytic function in AD, impairing neurotransmitter recycling and metabolic coupling between neurons and glial cells [17,34].

Major synaptic proteins, including synaptophysin, synaptotagmin, and PSD-95, are reduced in an AD patient's cortical tissue and cerebrospinal fluid (CSF), which is related to the severity and progression of the disease [32]. Several synaptic proteins are being investigated as molecular biomarkers for the early detection of synaptic compromise in neurodegenerative disorders [32]. In parallel, techniques to assess synaptic integrity in vivo are being developed, including positron emission tomography (PET) tracers targeting synaptic vesicle proteins [24]. Loss of synapses is strongly associated with memory impairment and also limits the brain’s capacity for plasticity and compensatory network reorganisation during neurodegeneration [8,33]. LTP and LTD process imbalance also occurs and fails to provide proper restructuring of the neural networks, which confines the scope of recovery [33]. Weakening of synaptic networks can disrupt cognitive processing by altering network balance and inducing compensatory hyperactivity in other brain regions [33]. Consequently, therapeutic interventions have increasingly focused on preserving synaptic health, with strategies aimed at enhancing synaptic resilience, reducing amyloid-beta (Aβ) accumulation, and stabilising tau protein to slow synaptic and cognitive decline [27,34]. There are also agents under study that enhance the expression of BDNF or regulate the glutamatergic transmission [34]. The strategies are also meant to delay or prevent the deterioration of synapses prior to the loss of neurons.

Role of synaptic transmission in major depressive disorder

Emotional, cognitive, and physical abnormalities are all linked to MDD, a common mental illness. The classical models focused on the inadequacy of monoamine neurotransmitters, including serotonin and norepinephrine, as the fundamental neurochemical causes of depression [25]. Although this framework has steered the innovations of selective serotonin reuptake inhibitors (SSRIs), it does not exhaust the explanation of the delayed onset of therapeutic effects and treatment resistance that is witnessed in a good proportion of the patients [26]. The more recent models concentrate on the synaptic connectivity and plasticity in MDD. One of the biggest risk factors for depression is stress, which lowers the density of the dendritic spine and the synaptic proteins in the hippocampus and prefrontal cortex [27]. These alterations weaken synaptic communication and contribute to deficits in mood regulation and cognitive flexibility [14,27].

Brain-derived neurotrophic factor (BDNF) is a neurotrophin that supports neuronal survival, synaptic growth, and activity-dependent synaptic plasticity, and, together with serotonin, serves as a key mediator of synaptic plasticity and neurogenesis [16]. In animal models, antidepressant treatments have been shown to raise BDNF levels, which are typically lower in MDD patients [28]. Tropomyosin receptor kinase B (TrkB) receptors are activated by BDNF, which contributes to the formation and maintenance of dendritic spines and increases synaptic strength [29]. One of the most significant advances in depression research is the recognition of ketamine’s rapid antidepressant effects. Ketamine is an antagonist of the NMDA receptor and produces clinical improvement within hours, in contrast to the delayed effects of conventional antidepressants [30]. Ketamine induces synaptic remodelling by increasing glutamate release and enhancing AMPA receptor-mediated transmission, thereby strengthening excitatory synaptic signalling. This process engages intracellular pathways such as the mammalian target of rapamycin (mTOR), a kinase that regulates protein synthesis and dendritic spine growth, ultimately promoting rapid synaptic strengthening and restoration of stress-induced synaptic deficits [31,32].

**Figure 3:**
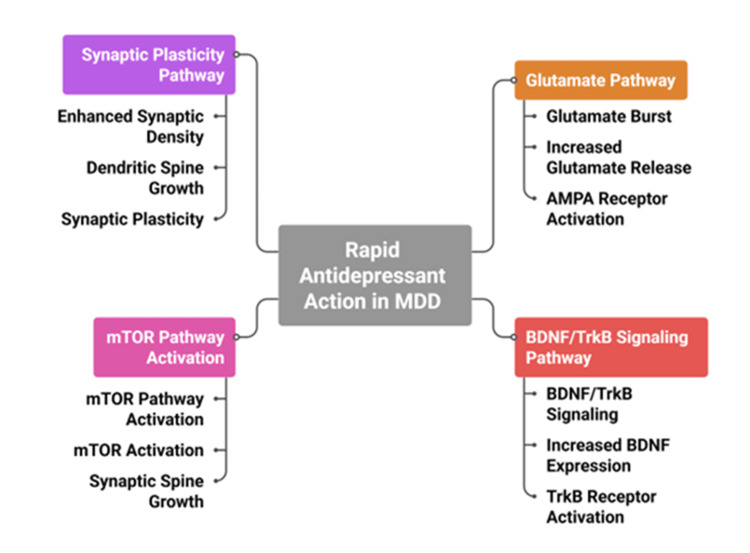
Mechanism of rapid antidepressant action in MDD. MDD: major depressive disorder; mTOR: mammalian target of rapamycin; AMPA: α-amino-3-hydroxy-5-methyl-4-isoxazolepropionic acid; BDNF: brain-derived neurotrophic factor; TrkB: tropomyosin receptor kinase B. The figure was created by the authors.

These findings have led to the development of novel pharmacological agents that preferentially target glutamatergic pathways rather than traditional monoaminergic systems, including NMDA receptor modulators, AMPA receptor potentiators, and ketamine-derived compounds such as esketamine, which exert rapid antidepressant effects [14,30,32]. In addition to neuronal mechanisms, glial cells play a significant role in the synaptic pathology of MDD [17]. Astrocytes are critical regulators of synaptic homeostasis, as they control extracellular glutamate levels through uptake mechanisms and provide metabolic support to neurons, and dysfunction of these processes contributes to synaptic instability and excitotoxic stress in depression [29,33]. The problem with astrocytic functioning in depression is that it becomes excitotoxic and prone to synaptic instability [33]. The activation of microglia, especially in chronic stress, facilitates the secretion of inflammatory cytokines, which exacerbate the synaptic signalling [34]. Functional neuroimaging research demonstrates that in depressed people, there is an unusual connectivity both inside and between networks related to mood. Such are the default mode, the salience, and executive networks, which require the integrity of synaptic transmission to facilitate their effective communication [35]. Dysregulation of these neural networks has been associated with symptoms such as rumination, reduced motivation, and impaired decision-making in MDD [35]. Figure 3 illustrates how a glutamate burst and activation of BDNF/TrkB signalling converge to enhance synaptic plasticity, thereby contributing to the rapid antidepressant effects observed in MDD [14,16,30].

Epilepsy and synaptic hyperexcitability

Epilepsy is a long-lasting neurological disorder that is manifested by frequent seizures due to hypersynchronous firing in the neurons. An essential process that gives rise to this pathology is the lack of balance between the excitatory and the inhibitory synaptic transmission [36]. GABA is the main inhibitory neurotransmitter in a healthy brain, which suppresses neuronal excitability by increasing the influx of chloride ions via GABAA receptors [37]. GABAergic inhibition is impaired in different epileptic syndromes either by loss of interneurons, by downregulation of GABA receptor subunits, or by reversal of the inhibitory gradient due to loss of chloride ion gradients [38]. This inhibitory decrease forms a lower threshold of action potential generation, making the excitatory inputs take over. Moreover, a range of epilepsy types are linked to GABA receptor subunit gene mutations, which interfere with the trafficking or functioning of the receptors [39].

With the excitatory side, glutamatergic transmission dysregulation contributes further to network excitability. The expression of AMPA or NMDA receptors is upregulated and their subunit composition distorted, which enhances calcium permeability and extends depolarisation [40]. There is also an increased probability of vesicle release and presynaptic facilitation that also contributes to excessive excitation, particularly in temporal lobe epilepsy [41]. Synaptic vesicle glycoprotein 2A (SV2A) is a presynaptic membrane protein involved in regulating synaptic vesicle exocytosis and calcium-dependent neurotransmitter release. It is present both in the excitatory and inhibitory terminals and has a role in vesicle priming and calcium sensitivity [42]. During loss or downregulation of expression of SV2A, the probability of release is changed, and synaptic stability is lost. A popular antiepileptic drug, levetiracetam, interacts with SV2A and restores normal cycling of vesicles, which decreases the number of occurrences of seizures [43].

Maladaptive synaptic reorganisation is a hallmark of chronic epilepsy. Structural alterations following recurrent seizures include mossy fibre sprouting in the hippocampus, a process in which axons of dentate granule cells abnormally grow and form recurrent excitatory synaptic connections within the dentate gyrus, thereby reinforcing hyperexcitable neural circuits and sustaining seizure activity [44]. Circuit instability is further exacerbated by dendritic spine remodelling and redistribution of synaptic contacts, structural changes that are particularly prominent in mesial temporal sclerosis, a hallmark pathology and major contributing factor in temporal lobe epilepsy [44]. In addition, astrocytic and microglial dysfunction contributes to synaptic hyperexcitability by impairing glutamate clearance, ionic homeostasis, and inflammatory regulation within epileptic networks [45,46]. Extracellular potassium and glutamate are normally buffered by astrocytes, but in the epileptic tissue, the manifestation of glutamate transporters is decreased, and therefore, the accumulation of glutamate at the synapses becomes excessive [45]. The microglia become activated and release inflammatory cytokines, which influence the expression of the receptors and raise the excitatory tone [46]. Genetic epilepsies are frequently linked to ion channel and synaptic protein mutations, including sodium voltage-gated channel alpha subunit 1 (SCN1A), which codes for a sodium channel component expressed in inhibitory neurons [47]. These mutations impair neuronal activity and increase susceptibility to seizures, and early therapeutic interventions targeting such genetic defects have shown promising results in preclinical models [47].

Parkinson’s disease: Dopaminergic synaptic disruption

Parkinson’s disease (PD) is a progressive neurodegenerative disorder characterised by motor manifestations, including bradykinesia, rigidity, and resting tremor [48]. The main pathological characteristic is the damage to dopaminergic cells of the substantia nigra pars compacta, which causes a significant decrease in the level of dopamine in the striatum [48]. Dopamine within the basal ganglia circuits acts as a key modulator that maintains the balance between the direct and indirect pathways of motor control. Activation of the direct pathway via dopamine D1 receptors facilitates movement, whereas activation of the indirect pathway through D2 receptors inhibits movement [49]. The decrease in dopaminergic tone enhances indirect route activity while decreasing direct pathway stimulation, which causes a decreased motor output [49].

PD is characterised by early synaptic dysfunction that precedes widespread neuronal loss [36,38]. One of the earliest pathogenic events is the accumulation of alpha-synuclein, a presynaptic protein involved in synaptic vesicle trafficking and SNARE complex assembly, which disrupts neurotransmitter release and synaptic integrity [36]. When misfolded, the alpha-synuclein aggregates block the docking of synaptic vesicles and decrease the efficiency of neurotransmitter release [36]. The accumulation of alpha-synuclein aggregates in the form of Lewy bodies represents a pathological hallmark of PD [36]. Another key process contributing to PD-related synaptic deficits is mitochondrial dysfunction [38]. Because dopaminergic neurons have high metabolic demands, mitochondrial impairment leads to synaptic energy deficits, compromising vesicle recycling, calcium buffering, and overall synaptic function [38,50]. This impairs ATP-dependent mechanisms, including vesicle recycling and calcium buffering, thereby compromising synaptic fidelity and increasing vulnerability to excitotoxicity, a pathological process in which excessive glutamate receptor activation leads to sustained calcium influx, neuronal damage, and synaptic loss [50].

Along with the loss of dopaminergic cells, an excessive glutamatergic activity is reported in the subthalamic nucleus, which leads to the formation of inappropriate burst firing and oscillations in basal ganglia loops [38]. Such perturbations negatively affect the time accuracy of motor output, and they may be the basis of symptoms that include tremor and bradykinesia. The circuitry is also unbalanced in globus pallidus and substantia nigra reticulata, where GABAergic changes are present [39]. Neuroinflammation adds another layer of complexity. The cytokines excreted by the activated microglia in the case of PD (tumour necrosis factor-alpha (TNF-α) and IL-1beta) inhibit the synaptic plasticity and encourage the death of neurons [40]. Excitotoxic stress is also caused by astrocytic dysfunction, such as the loss of dopamine uptake, and decreased glutamate clearance [41].

Aberrant synaptic activity in PD can be partially restored through deep-brain stimulation (DBS), a neurosurgical intervention in which electrodes are implanted in motor-related nuclei such as the internal globus pallidus or subthalamic nucleus to deliver controlled electrical stimulation. DBS improves motor function by modulating abnormal neural activity, and although its precise mechanisms are not fully understood, it is thought to alter local field potentials and reduce pathological synchronisation within basal ganglia circuits, thereby restoring more physiological patterns of motor signalling [42]. New treatment modalities are examining how to guard or repair synaptic activity. These are alpha-synuclein aggregation inhibitors, mitochondrial protectants, and gene therapy that increase the production of dopamine or transport it [43]. There is also a need to study the GABAergic and glutamatergic transmission modulators as a way of managing symptoms and neuroprotection [44].

Microglia-synapse interaction in neuroinflammation

Microglia, the resident immune cells of the central nervous system, play an essential role in maintaining synaptic homeostasis through activity-dependent surveillance and synaptic regulation [46]. They also constantly scan the neural landscape and are active in development and disease, in activity-dependent synaptic pruning [46]. Molecular signals, including complement protein components of the innate immune system that tag cellular elements for removal and patterns of neuronal activity, regulate microglial involvement in synaptic remodelling. Complement proteins C1q and C3 are selectively deposited on less active or weakened synapses, where they act as molecular “tags”. These tagged synapses are recognised by microglial complement receptor 3 (CR3), triggering complement-dependent synaptic engulfment and elimination. The mechanism is necessary in brain maturation, yet it may be maladaptive during neurodegenerative or inflammatory diseases [47]. In AD, as another example, activation of complement results in over-pruning of functional synapses, which promotes early mental impairment [48].

Microglial activation can be triggered by a wide range of stimuli, including neural injury, infection, and chronic stress [31,48]. Upon activation, microglia release pro-inflammatory cytokines, such as interferon-gamma (IFN-γ), TNF-α, and interleukin-6 (IL-6), which modulate synaptic plasticity and alter neurotransmitter receptor trafficking, thereby influencing synaptic function and network stability [31,49]. These mediators interfere with the homeostasis between glutamatergic and GABAergic and destabilise the dendritic spine [49]. Constant exposure to these signals worsens the LTP and increases the degeneration of the synapses. In multiple sclerosis, microglial activity has been shown to cause synaptic loss independent of demyelination [18,50]. Investigations reveal that microglia actively engulf synaptic components in both white and grey matter, and this is linked to patients' cognitive impairment [50]. Cytokines and signalling molecules released by astrocytes, including interleukin-33 (IL-33) and adenosine triphosphate (ATP), regulate microglial synaptic pruning by modulating microglial activation thresholds and responsiveness [17,31].

Chronic stress and elevated glucocorticoid levels in MDD promote microglial sensitisation [12,31]. In brain regions such as the hippocampus and prefrontal cortex, activated microglia adversely affect synaptic connectivity and neurogenesis, contributing to mood and cognitive dysfunction [12,29]. These are accompanied by a decline in motivation and anhedonia as well as cognitive blunting [12]. A causal relationship is supported by evidence demonstrating that several stress- and inflammation-induced synaptic alterations can be reversed by anti-inflammatory interventions in animal models [29,31]. In addition, microglia actively regulate synaptic remodelling through direct physical contact, extending fine processes toward individual synapses and rapidly modulating synaptic stability in response to changes in neuronal activity [19,46]. This fine-tuned modulation contributes to experience-dependent plasticity and learning [19]. Therapeutically, targeting microglia-synapse interactions has emerged as a growing area of interest in neuroinflammatory and neurodegenerative disorders [31,46]. Proposed strategies include complement pathway inhibitors, controlled microglial depletion followed by repopulation, and anti-inflammatory agents designed to preserve essential microglial surveillance while limiting excessive synaptic pruning [31,34]. Achieving this balance is critical to prevent disruption of normal immune functions within the central nervous system while minimising pathological synaptic loss [46]. Table 2 summarises key microglial mechanisms affecting synapses, their triggers, molecular mediators, disease associations, synaptic effects, therapeutic strategies, and references.

**Table 2:** Microglial regulation of synapses in health and disease. C1q: complement component 1q; C3: complement component 3; CR3: complement receptor 3; IL-6: interleukin-6; TNF-α: tumour necrosis factor-alpha; IFN-γ: interferon-gamma; MDD: major depressive disorder; PFC: prefrontal cortex; IL-33: interleukin-33; ATP: adenosine triphosphate; MS: multiple sclerosis; BDNF: brain-derived neurotrophic factor; LTP: long-term potentiation; E/I: excitation-inhibition.

Process	Stimuli	Molecular players	Disease associations	Synaptic effects	Therapeutic strategies	Reference number
Surveillance & Synaptic Pruning (Normal)	Neuronal activity: developmental signals	C1q, C3, CR3	Brain development	Removes weak synapses; shapes circuits	Not applicable (physiological pruning - no intervention required)	[46]
Pathological Over-Pruning	Excess complement activation	C1q, C3, CR3	Alzheimer’s disease	Loss of functional synapses; cognitive decline	Complement inhibitors	[48]
Pro-inflammatory Activation	Infection, injury, and chronic stress	IL-6, TNF-α, IFN-γ	MDD, neurodegeneration	E/I imbalance; dendritic spine loss; reduced plasticity	Anti-inflammatory agents	[29]
Chronic Microglial Sensitisation	Elevated glucocorticoids (chronic stress)	Glucocorticoid receptors; cytokine signalling	MDD	Decreased neurogenesis, PFC & hippocampal connectivity	Glucocorticoid modulators, anti-inflammatories	[12]
Astrocyte–Microglia Crosstalk	Astrocyte activation	IL-33, ATP	MS, MDD	Adjusts pruning thresholds; may cause excessive synaptic loss	Targeting astrocytic signalling (e.g., IL-33 blockade)	[20]
Synapse Engulfment Without Demyelination	Chronic inflammation	CR3, C1q, C3	Multiple sclerosis	White & grey matter synapse loss; cognitive impairment	Microglial depletion/repopulation	[34]
Experience-Dependent Remodelling	Neuronal activity (real-time)	Physical microglial contact	Learning and plasticity (physiological)	Spine remodelling: dynamic plasticity	Not applicable (normal adaptive mechanism)	[19]
Reversal of Synaptic Damage	Anti-inflammatory interventions	BDNF, anti-inflammatory cytokines, and synaptic scaffolding proteins	MDD, animal models	Recovery of LTP and synaptic density	Targeted anti-inflammatory or synaptic repair agents	[32]

Therapeutic approaches targeting synaptic mechanisms

The emergence of therapies that directly target synaptic processes has been the result of the increasing appreciation of synaptic dysfunction as an important disease in numerous mental and neurological conditions. Such approaches involve drugs, genetic, and circuitry-based manipulations [36]. Pharmacological treatments modulate receptor function or neurotransmitter availability. The NMDA receptor antagonist, such as memantine, limits the glutamate excitotoxicity of AD by attenuating the influx of calcium in response to pathological stimulation [37]. SSRIs increase the serotonergic tone in depression and have indirect effects in stimulating synaptic plasticity by downstream actions on BDNF expression [38]. Other compounds directly affect synaptic proteins. Levetiracetam also controls the synaptic vesicle cycling by binding the SV2A and inhibiting the aberrant neurotransmitter release in epilepsy [29]. Ampakines are positive allosteric modulators of AMPA receptors, which improve excitatory transmission, and were found to have cognitive effects in preclinical studies [10].

The rationale behind these therapeutic approaches is to enhance synaptic strength and function rather than merely altering neurotransmitter levels [2,14]. Gene therapy offers a more precise strategy by targeting the underlying genetic defects responsible for impaired synaptic function, thereby addressing the root cause of synaptic dysfunction in neuropsychiatric and neurodevelopmental disorders [41]. Clustered regularly interspaced short palindromic repeats-CRISPR-associated protein 9 (CRISPR-Cas9) is a genome editing technology that enables precise, targeted modification of DNA sequences, allowing correction or disruption of disease-causing genetic mutations. Using CRISPR-Cas9, it is possible to rectify pathogenic mutations in genes such as methyl-CpG-binding protein 2 (MECP2), which is implicated in Rett syndrome, and fragile X messenger ribonucleoprotein 1 (FMR1), the causative gene in fragile X syndrome, thereby offering a potential strategy for durable correction of synaptic dysfunction [41]. Such approaches remain in their early stages but show promise for achieving durable correction of synaptic dysfunction [41]. Chemogenetics and optogenetics are neuromodulation techniques that enable precise spatial and temporal control of neuronal activity: chemogenetics uses engineered receptors activated by designer drugs to modulate neuronal firing, whereas optogenetics employs light-sensitive ion channels to control neuronal activity with high temporal precision [13,42]. Designer drugs can modulate chemogenetic receptors such as designer receptors exclusively activated by designer drugs (DREADDs), which are engineered G protein-coupled receptors that allow selective augmentation or suppression of synaptic transmission within specific neural circuits [42].

Optogenetics is a neuromodulation technique that involves the genetic introduction of light-sensitive ion channels or pumps into specific neurons, allowing their activity to be precisely controlled using light. This approach enables real-time investigation and potential intervention in dysfunctional neural circuits in disorders such as PD and depression [13]. The use of precision medicine involves stratification of patients in terms of molecular and functional synaptic markers. PET tracers of synaptic density or CSF synaptophysin levels can be used as biomarkers to identify individuals with early synaptic pathology [24]. The selection of treatment can be made using the genomic profiling based on patterns of gene expression related to synapses, which enhances the heterogeneous disorder response rates [35]. Challenges remain in targeting synapses effectively. Important issues are medication distribution across the blood-brain barrier to the brain, long-term safety of gene editing, and the necessity to strike a balance between synaptic strengthening and the danger of excitotoxicity. However, the emphasis on restoring the synapses is an indication of a transition from symptomatic and disease-modifying treatment. A comparative summary of the major synaptic alterations, molecular mechanisms, and therapeutic implications across the reviewed neurological and psychiatric disorders is presented in Table 3.

**Table 3:** Summary of synaptic alterations across neurological and psychiatric disorders. ASD: autism spectrum disorder; NMDA: N-methyl-D-aspartate; FMRP: fragile X mental retardation protein; DISC1: disrupted-in-schizophrenia 1; ErbB4: erythroblastic leukaemia viral oncogene homolog 4; SV2A: synaptic vesicle glycoprotein 2A; MDD: major depressive disorder; BDNF: brain-derived neurotrophic factor; AMPA: α-amino-3-hydroxy-5-methyl-4-isoxazolepropionic acid; GABA: gamma-aminobutyric acid; SCN1A: sodium voltage-gated channel alpha subunit 1; PD: Parkinson’s disease; D1/D2: dopamine receptor D1 and dopamine receptor D2.

Disorder	Major synaptic alterations	Key molecular/synaptic players	Predominantly affected brain regions	Functional consequences	Therapeutic implications
ASD	Disrupted synaptic adhesion; altered excitation–inhibition balance	Neurexins, neuroligins, FMRP	Cortex, hippocampus	Impaired circuit maturation and synaptic selectivity	Synapse-stabilising and E/I-balancing strategies
Schizophrenia	NMDA receptor hypofunction; impaired synaptic plasticity	NMDA receptors, DISC1, ErbB4	Prefrontal cortex, hippocampus	Cognitive dysfunction and working memory deficits	Glutamatergic modulation; synaptic plasticity enhancement
Alzheimer’s disease	Synaptic loss, impaired vesicle cycling, and receptor trafficking	Amyloid-β, Tau, SV2A	Hippocampus, association cortex	Memory loss and reduced network adaptability	Synapse-protective and disease-modifying therapies
MDD	Reduced synaptic density; impaired plasticity	BDNF, AMPA receptors	Prefrontal cortex, hippocampus	Mood dysregulation and cognitive inflexibility	Rapid-acting antidepressants targeting synaptic plasticity
Epilepsy	Hyperexcitable synaptic networks; maladaptive reorganisation	GABA receptors, SCN1A	Hippocampus (mesial temporal lobe)	Recurrent seizures and network instability	Circuit stabilisation and synaptic inhibition enhancement
PD	Dopaminergic synaptic dysfunction; altered basal ganglia circuitry	α-synuclein, D1/D2 dopamine receptors	Basal ganglia	Motor impairment and abnormal synchronisation	Deep brain stimulation and synaptic modulation

Limitations and future recommendations

Although the field of molecular neuroscience has witnessed substantial advances, this narrative review is subject to several inherent methodological limitations. As a non-systematic synthesis of the literature, this review relies on qualitative interpretation of existing studies and may be influenced by publication bias, selective reporting, and the absence of predefined inclusion, exclusion, or quantitative weighting criteria, thereby limiting the ability to draw definitive causal inferences or perform comparative effect estimations across studies [27,36]. In addition, much of the evidence summarised is derived from rodent models of disorders such as ASD and AD, which do not fully capture the cognitive complexity, genetic heterogeneity, and environmental variability observed in human populations [15,36]. Species-specific differences in synaptic architecture, neurotransmission, and behavioural phenotypes further constrain the direct translation of these findings to clinical contexts [15].

Another important limitation is the incomplete understanding of the dynamic interactions between genetic susceptibility, epigenetic regulation, and environmental exposures. For instance, the mechanisms through which prenatal stress interacts with synaptic gene variants to alter neurodevelopmental trajectories in schizophrenia remain poorly defined [35]. Moreover, many studies focus on molecular events at individual synapses, with limited integration across neural circuits or behavioural outcomes, an issue compounded by the scarcity of longitudinal human data examining synaptic structure and function across the lifespan, including development, ageing, and disease progression [19,27]. Despite these limitations, a major strength of this narrative review lies in its integrative and cross-diagnostic perspective. By synthesising molecular, cellular, imaging, and translational findings across multiple neurological and psychiatric disorders, this review highlights shared synaptic vulnerabilities and convergent mechanisms that may not be evident in disorder-specific analyses [8,14]. This systems-level approach supports the conceptualisation of synaptic dysfunction as a unifying framework for understanding diverse brain disorders and identifying common therapeutic targets.

Future research should therefore adopt integrative, multi-scale strategies that bridge molecular biology with systems neuroscience, advanced neuroimaging, and computational modelling to better capture how synaptic alterations propagate through brain networks to influence behaviour [27,36]. Emerging precision neuroscience tools, including single-cell RNA sequencing and spatial proteomics, hold promise for identifying disease-specific synaptic signatures and enabling patient stratification for personalised interventions [16]. Translational pipelines should also prioritise biomarker validation studies linking synaptic dysfunction to clinical symptoms using fluid-based assays and functional imaging [24]. Therapeutic innovation may increasingly focus on synapse-targeted delivery of gene therapies or neurotrophic factors using nanocarrier systems, several of which are currently under investigation in early-phase trials for mood and neurodegenerative disorders [41]. Finally, longitudinal, lifespan-focused studies are essential to identify periods of heightened vulnerability or recovery, thereby advancing a more clinically relevant understanding of synaptic biology in brain health and disease [19].

## Conclusions

This narrative review synthesises current evidence on the role of synaptic transmission in the pathophysiology of major neurological and psychiatric disorders, presenting a systems-level framework that spans 10 key domains of synaptic dysfunction. By emphasising shared molecular and network-level vulnerabilities across conditions such as schizophrenia, ASD, AD, epilepsy, and MDD, the review positions synaptic dysfunction as a unifying mechanism underlying diverse brain disorders. By integrating mechanistic insights with translational relevance, this review highlights emerging synaptic biomarkers, molecular regulators, and circuit-level tools that are shaping advances in diagnosis and therapy. The findings underscore the value of multimodal, precision-oriented approaches that connect molecular changes to circuit dysfunction and clinical outcomes, thereby providing a coherent foundation for future research and synapse-targeted therapeutic strategies.
